# Supplementary material for: Novel Synergistic Approach for Bioactive Macromolecules: Evaluating the Efficacy of Goat Placenta Extract in PEGylated Liposomes and Microspicules for Chemotherapy-Induced Hair Loss
Source: Pharmaceuticals (Basel). 2024 Aug 19;17(8):1084. doi: 10.3390/ph17081084 (PMC11360673; doi:10.3390/ph17081084)
Supplement: Supplementary file 1 [file pharmaceuticals-17-01084-s001.zip › pharmaceuticals-3131833-supplementary.pdf]

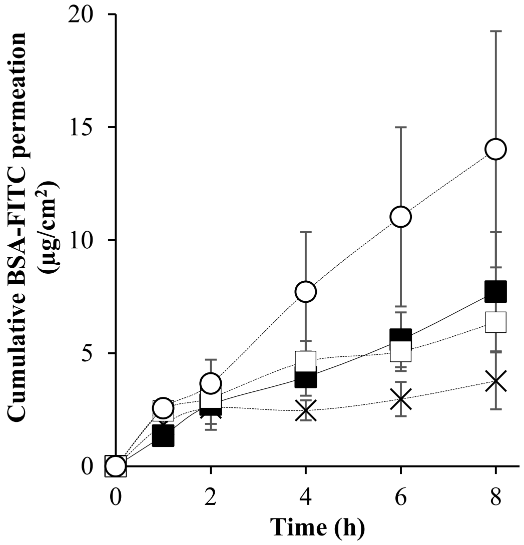

Figure S1: Cumulative permeation of BSA-FITC versus time profiles of PL (○), gel (■), MS gel (□) and solution (×). The data presents the mean ± S.D. (N = 3).
